# Outfitting the quest for spatial spread of infections: A review of mobility datasets for population health modelling

**DOI:** 10.1016/j.idm.2026.03.006

**Published:** 2026-04-01

**Authors:** Nodira Tillayeva, Naoki Tamura, Kenta Urano, Takuro Yonezawa, Nobuo Kawaguchi, Takashi Okumura

**Affiliations:** a Graduate School of Engineering, Nagoya University, Nagoya, Japan; b Institutes of Innovation for Future Society, Nagoya University, Nagoya, Japan; c Kitami Institute of Technology, Kitami, Japan

**Keywords:** Human mobility, Infectious disease, Synthetic data, Public health modeling, GPS datasets, Mobility networks

## Abstract

Understanding the spatial dynamics of infectious disease spread is essential for modeling population health. A key component in such modeling is human mobility data, which informs how infections propagate across time and space. This review provides a comprehensive survey of both real-world and synthetic mobility datasets that have been used in the context of infectious disease modeling. Through the survey, we identified 57 publicly available datasets—52 real-world and 5 synthetic—offering a structured overview of current data sources. Additionally, because real-world data are often inaccessible due to privacy or technical constraints, we provide a concise overview of pseudo-mobility data generation methodologies to contextualize the synthetic datasets and guide future data-production efforts. The review highlights the need to advance synthetic data generation methodologies and improve the accessibility of high-resolution mobility data for future research in this domain.

## 1 Introduction

To elucidate the dynamics of infectious disease transmission, it is crucial to analyze human mobility and behavior. For instance, to simulate the spread of an infection in a given population, information about who moved where, when, and for how long is indispensable. Since the onset of the COVID-19 pandemic, the attention to such infectious disease simulations has increased, highlighting the importance of human mobility data (Fan et al., 2022; Zachreson et al., 2021). Governments that implemented measures such as lockdowns and travel restrictions could better understand how these interventions reduced contact opportunities and helped contain disease spread (Yabe et al., 2020), by analyzing the movement data.

With sufficiently comprehensive and high-quality movement data, one can analyze how infectious diseases spread due to human movement and behavior, and predict the potential scope of future outbreaks. Accurate and high-resolution mobility data enable policymakers to identify high-risk areas promptly, allocate limited resources effectively, and design timely interventions to mitigate large-scale outbreaks. Improving the fidelity of mobility-based simulations can substantially benefit both public health and society at large. However, repositories for such data have not been well established, and previous studies that have conducted surveys in this field are often incomplete or outdated, which underscores the need for an updated and structured review (Hu et al., 2021).

The data pertinent to human mobility and geographic location can be categorized into two primary classes: real data recorded from the actual movements of residents, and *pseudo-data* (often referred to as synthetic data), which is generated by simulating human mobility patterns based on real-world data input. The former, while providing fidelity to actuality, faces issues related to the costs associated with acquisition, the resolution of spatio-temporal data, population coverage, and concerns regarding individual privacy. In contrast, pseudo-data offers wider coverage and better privacy protection but faces challenges with reliability and reproducibility. The provenance of such data profoundly influences the validity of simulations, while the resolution and volume of data also impose constraints on the range of simulation modalities attainable.

Traditionally, such data was derived from person-trip surveys, national censuses, and other large-scale transportation studies. However, with the widespread use of mobile devices and the development of Internet of Things (IoT) technologies, an increasingly rich and diverse stream of location and movement data is now available. To define the paper's scope at this stage, we present three use cases for each data resolution level.

**Administrative Division Level** Correlation analysis can be performed between mobility in administrative divisions, such as states or provinces, and the spread of infections (Chen, Zhang, et al., 2020; Fraiberger et al., 2020; Jeffrey et al., 2020). These analyses are beneficial for making policy decisions on movement restrictions or lockdowns at the administrative level, e.g., state governments or local authorities.
**Mesh Area Level** Infection risks can be quantified and mapped at the mesh level by dividing regions into grids (Kimura et al., 2023). Such infection risk maps offer valuable information not only for decision-making in infection control measures, such as regulations for specific areas, but also for individual infection prevention.
**Individual and Facility Level** With spatially and temporally high resolution data, more detailed modeling of infectious diseases becomes feasible, enabling strategies to be devised to prevent disease spread from its initial stages (Fan et al., 2022; Huang et al., 2020; Li, Huang, Ye, et al., 2021). For this purpose, detailed movement trajectories of individuals, their locations of stay, and time information should be acquired at the point level in terms of latitude, longitude, and timestamp.

In the following sections, we first provide the survey methodology. In this process, we focus on datasets that meet two key criteria: (i) a temporal resolution of daily or finer and (ii) a spatial resolution at the city or neighborhood level. These thresholds are critical for capturing epidemiologically meaningful mobility patterns, such as daily commuting flows that drive local transmission and cross-regional trips that contribute to long-distance spread. Datasets aggregated at coarse levels (e.g., monthly or nationally) are excluded because they lack the fidelity needed to resolve transmission mechanisms. Section 3 outlines the dataset found, in both the real-world data and the synthetic (pseudo) mobility datasets. However, because there are a limited number of pseudo datasets that are publicly available, we also reviewed methodologies for the generation of human mobility dataset for future infectious disease modeling. Section 4 provides a discussion on the survey results, and finally, Section 5 concludes the paper.

## 2 Methods

**Fig. 1:**
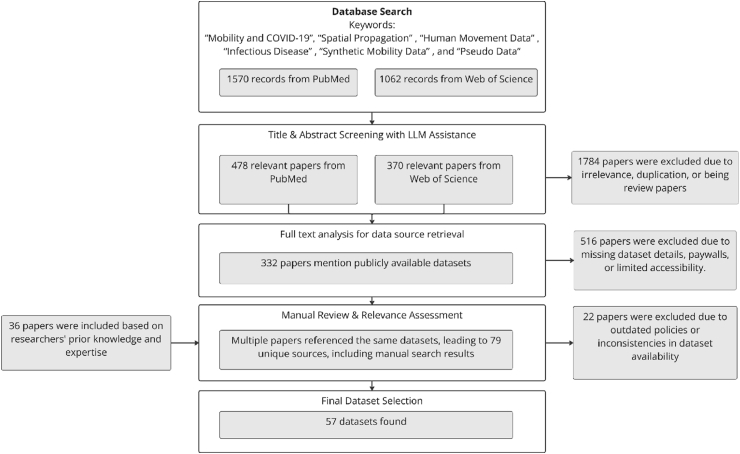
Flow diagram illustrating the dataset identification, screening (with LLM assistance), and selection process—from initial *PubMed* and *Web of Science* searches to the final inclusion of 57 publicly available datasets.

We conducted a systematic literature search using *PubMed* and *Web of Science*, identifying English-language articles published between January 2010 and December 2025 that address human mobility in relation to infectious disease spread. The goal of this study was to investigate the current status of human mobility data relevant to infectious disease transmission modeling and to summarize available datasets that meet daily or finer temporal resolution and city- or neighborhood-level spatial granularity. Our protocol is outlined in Fig. 1.

For both *PubMed* and *Web of Science*, we applied a consistent Boolean search query combining mobility-related, disease-related, and modeling-related terms. The search terms are summarized in Table 1. We applied English-language and date filters (January 2010–December 2025), prioritized open-access articles where available, and excluded review or survey papers during screening. Please note that this review was designed to capture as many relevant datasets and methodologies as possible, rather than follow a strict scoping review protocol. Accordingly, searches in *Web of Science* were conducted using its interactive keyword interface, to expand the search scope interactively to refine results and maximize coverage. The final search was conducted on 23 February 2026, yielding 1570 records from *PubMed* and 1062 from *Web of Science*.

**Table 1:** Boolean search query structure.

Search Dimension	Terms
Mobility-related terms	“mobility data” OR “mobile phone data” OR “human movement data” OR “human mobility” OR “location data” OR “GPS data” OR “mobility report”
Disease-related terms	“infectious disease” OR “disease spread” OR “COVID-19″ OR “SARS-CoV-2″ OR “epidemic” OR “pandemic”
Modeling-related terms	“synthetic data” OR “pseudo-data” OR “simulated data” OR “agent-based model” OR “mobility modeling”

By design, our primary search targeted epidemiological and public-health databases (*PubMed* and *Web of Science*), as these are the principal venues for studies that apply mobility datasets to infectious disease modeling—the core focus of this review. Likewise, the search terms were intentionally centred on mobility *data* rather than generation algorithms, using broad keywords so that the LLM-assisted screening could organically surface related methodological contributions alongside dataset descriptions. Computer-science venues (e.g., IEEE Xplore, ACM Digital Library), where recent trajectory-generation and OD (Origin Destination)-estimation methods are primarily published, were covered through a supplementary manual survey and citation chaining; the pseudo-data generation methods discussed in Section 3.4 were identified through this complementary pathway.

A large language model (LLM)-based classifier was then applied to screen for relevance, removing irrelevant entries, duplicates, and review articles. Specifically, the classifier was applied to the titles and abstracts of all retrieved records and assigned a binary relevance label based on predefined inclusion criteria (focus on human mobility and infectious disease spread, with sufficient methodological detail). Duplicates were identified and removed by comparing metadata fields such as title, DOI, and publication year. Records flagged as reviews were excluded by detecting characteristic keywords (e.g., “review,” “survey”) in titles or abstracts. After this automated stage, all articles labeled as relevant were manually checked for confirmation before proceeding to full-text screening. This process excluded 1784 articles, leaving 478 relevant papers from *PubMed* and 370 from *Web of Science*.

Next, we performed full-text screening to identify studies referencing publicly available mobility datasets with sufficient spatiotemporal resolution—defined as: (i) temporal granularity of at least daily intervals and (ii) spatial resolution at the city or neighborhood level. These thresholds were chosen to ensure that fine-grained mobility patterns relevant to local transmission dynamics could be captured. While city-level data may be ideal for dense urban areas, county-level or regional data may suffice for sparsely populated regions.

Full texts were retrieved using automated web searches targeting reputable academic repositories. These documents were then analyzed by a fine-tuned LLM to extract information on dataset availability, resolution adequacy, and access conditions. As a result, 332 papers were retained. The remaining 516 were excluded due to lack of dataset references, insufficient resolution (e.g., monthly or nationally aggregated data), or limited accessibility (e.g., behind paywalls).

Then, a manual review revealed that many articles referred to overlapping datasets. This consolidation resulted in 79 unique dataset sources. Twenty-three of these were excluded because the information was outdated or access had been revoked. To supplement our findings, 36 additional papers were included based on preliminary survey. In total, 57 publicly available datasets met the selection criteria of (i) daily or finer temporal resolution and (ii) city- or neighborhood-level spatial granularity.

These datasets enable a range of applications. High-resolution GPS data (e.g., anonymized smartphone traces) can support real-time detection of mobility hotspots. Meanwhile, synthetic models allow “what-if” simulations for emerging diseases. Even lower-resolution data (e.g., airline passenger flows) remain useful for analyzing inter-regional transmission risks.

While the specific threshold for “sufficient resolution” depends on local epidemiological context, capturing at least daily changes and subregional (e.g., city or county) spatial granularity generally ensures useful insights. The review process described above ensures that the datasets included are both accessible and sufficiently detailed for effective infectious disease modeling.

## 3 Results

### 3.1 Open real-world mobility data

First, we consolidate publicly available movement data that can be utilized for a variety of tasks, starting with the analysis of infectious disease propagation. These datasets are freely provided by local governments, research institutions, and companies, and are available for research purposes. Here, we categorize the data into four categories: (1) Administrative region level data, (2) Mesh (grid) level mobility data, (3) Origin-destination trip data, and (4) Point-level movement data. A list of the datasets is presented in Table 2a, Table 2ba and 2b.

**Table 2a:** Summary of publicly available human mobility datasets (Part 1: administrative-level and mesh-level data).

Data Category	Name & Provider	Region	Period	Spatial Resolution/# Locations	Temporal Resolution/# Records
Admin. Division Level Statistics and Index Data	Google LLC∗∗	Worldwide	2020/02/15–2022/10/15	City, Country, State	1 Day
	Apple Mobility Data∗∗	Global	2020/01/13–2022/04/14	City, Region, Country	1 Day
	Baidu Mobility Index	China	2020/03/15–present	City, Province	1 Day
	Descartes Lab Mobility Index	U.S.	2020/03/01–2021/04/20	State, Country	1 Day
	University of Maryland Mobility Metrics	U.S.	2020/01/01–2021/04/20	State	1 Day
	ODT Flow Explorer	U.S.	2019/01/01–2021/04/15	State, Country	1 Day
	Tencent Migration Data Qianxi	China	2020/01/01–present	City, Province	1 Day
	SafeGraph Social Distancing Metrics∗∗ (now Advan)	U.S.	2020/03/01–2022/12/31	State, County	1 Day
	Cuebiq Data for Good	Global	2020/01/01–present	Administrative Divisions	1 Day
	Meta Data for Good	Global	2020/01/01–present	Administrative Divisions	1 Day
	BBC Pandemic Model Data	UK	2018/01/01–2019/12/31	City, Region	Survey Period
	GLEAMviz H1N1 Mobility	Global	2009/01/01–2010/12/31	Country	Modeled Daily
	Unacast Social Distancing Dataset∗∗	Global	2020/03/01–2021/12/31	Administrative Divisions	1 Day
	MobMeter (Finazzi/Univ. Bergamo)	17 countries	2020/03–present	Country, Subnational	1 Day
Mesh Level Mobility Data	Telecommunications Data	Milan, Italy	2013/11/01–2014/01/01	250 m Mesh	Hourly
	WorldPop Dynamic Population	France, Portugal	2007/05/01–2007/10/31	100 m Mesh	1 Day
	Yahoo JAPAN Congestion Ladder	Japan	Historical (1 Day)	100 m Mesh	Hourly
	NTT docomo Mobile Spatial Statistic	Japan	Historical (1 Day)	500 m Mesh	Hourly
	D4D Orange Telecom	Côte d’Ivoire, Senegal	2013/01/01–2013/12/31	500 m Mesh	1 Day
	LYMob-4Cities	Japan	2020/01/01–present	100 m Mesh	Hourly
	dtac Mobility Data	Thailand	2020/03/01–present	1 km Mesh	1 Day
	YJMob100K (Yahoo Japan/LY Corp.)	Japan	75 days (60 + 15 emergency)	500 m Mesh	30 min; 100K users

For full dataset references and providers, see Supplementary Table A1.

∗∗ Datasets have ceased updating or are no longer publicly available.

**Table 2b:** Summary of publicly available human mobility datasets (Part 2: OD trip and point-level data).

Data Category	Name & Provider	Region	Period	Spatial Resolution/# Locations	Temporal Resolution/# Records
OD Trip Data	CMAP Travel Survey	U.S.	2017/10/02–2019/01/20	110 k Locations	98 k Records
	MITMA Daily OD	Spain	2020/01/01–present	Municipalities	1 Day
	NYC Taxi Trips	NYC, U.S.	2009/01/01–present	Taxi Zones	Trip-based
	Chicago Divvy Bike	Chicago, U.S.	2013/06/01–present	Stations	Trip-based
	DiDi GAIA	Chengdu, China	2018/11/01–2018/11/30	Road Segments	2–4 s
	Global Airline Traffic	Global	2019/01/01–present	Airports	1 Day
	MOBINS-Epidemic-Korea (KAIST DMLAB)	Korea (Seoul, Busan, Daegu)	COVID-19 pandemic period	City, District	1 Day
	MOBINS-Epidemic-NYC (KAIST DMLAB)	NYC, U.S.	COVID-19 pandemic period	Borough, Zip	1 Day
	US Census LODES/LEHD	U.S.	2002–present (annual)	Census Block, Tract	Annual; ∼7M OD pairs
Point-Level Movement Data	AnLoCOV	Ecuador	2013/06/21–2022/04/05	Point (Latitude, Longitude)	16M Records
	MOBIS	Switzerland	2019/09/01–2020/02/29		3M Records
	Shanghai GPS Data	Shanghai, China	2013/10/18–2015/11/25		10k Records
	NREL California Survey	California, U.S.	2012/02/01–2013/02/03		3M Records
	CRAWDAD EPFL Taxi	San Francisco, U.S.	2008/05/17–2008/06/10		112M Records
	Copenhagen Networks Study	Denmark	2013/01/01–2014/12/31		1M Records∗
	MIT Reality Mining	Boston, U.S.	2004/09/01–2005/06/30		1M Records∗
	Geotagged Tweets	Global	2020/01/01–present		1B + Records
	OpenStreetMap GPS	Global	2005/01/01–present		50M Records∗
	Iquitos GPS	Peru	2018/01/01–2019/12/31		150k Records
	LBSLab WeChat	China	2020/03/01–present		10M Records
	Geolife GPS Trajectories	Beijing, China	2007/04/01–2012/08/31		1–5 Seconds
	CRAWDAD ROMA/TAXI	Rome, Italy	2014/02/01–2014/03/02		7 Seconds
	ECML/PKDD 15 Taxi	Porto, Portugal	2013/01/07–2014/06/30		15 Seconds
	TaxiBJ21	Beijing, China	2012/11/01–2015/11/30 (3 months)		1 Minute
	Mobile Data Challenge	Switzerland	2009–2011		1–5 Seconds
	T-Drive	Beijing, China	2008/02/02–2008/02/08		15M Records
	Brightkite Check-in	Global	2008/04/01–2010/10/31		4.5M Records
	Gowalla Check-in	Global	2009/02/01–2010/10/31		6.4M Records
	GeoCOV19Tweets	Global	2020/03/20–2023/03/15		502k Records
	MegaGeoCOV	Global	2020/01/01–2021/09/16		25M Records

For full dataset references and providers, see Supplementary Table A1.

∗ Approximate values.

Administrative-level datasets record indexed statistics such as intra-regional movement and inflow/outflow across administrative boundaries. From these datasets, one can analyze inter-city mobility and the intensity of human movements within cities, conducting correlation analyses with the spread of infectious diseases. However, due to low spatial and temporal resolution, it is difficult to perform analyses at the individual level or within fine city sectors.

Mesh-level mobility data offers finer spatial granularity than administrative-level data and provides a larger data volume than most point-level datasets. However, because the temporal resolution ranges from hourly to daily, it is difficult to reconstruct detailed individual trajectories. Additionally, geographic availability is more limited compared to administrative datasets.

Origin–Destination trip data captures mobility flows between fixed points (e.g., transit stations), including trip volumes. These datasets enable point-to-point movement tracing and can support more detailed infectious disease modeling. However, for propagation modeling, not just movement but also dwell time or stay information is required. Therefore, OD trip data alone is insufficient for modeling contact-based transmission mechanisms.

Lastly, point-level movement data collected via GPS enables tracking of individual movements and stays, offering significant utility for infectious disease modeling. While positioning errors pose challenges, it can help estimate individual contact probabilities (Fan et al., 2022). However, many of these datasets are not publicly available due to privacy concerns and high collection costs. Even when available, they often suffer from sparse sampling (e.g., one point every few hours) or are limited to specific users or regions, such as taxis (Bracciale et al., 2022; Moreira-Matias et al.; Jiang, 2022; Piorkowski et al., 2022). Thus, the utility of such data for city-wide propagation modeling is constrained by limited spatial and temporal coverage.

Although publicly available mobility data is easily accessible, it is often too coarse or delayed for detailed infectious disease modeling. Effective modeling requires data that are timely enough to support forecasts days or even months in advance. During the peak of the COVID-19 pandemic (2020–2022), many companies released their location data for public use. Although some datasets have since been made private, we retain them in our dataset table as references.

### 3.2 Proprietary mobility data: capabilities and caveats

Mobility data collected from mobile apps by private companies exists as proprietary data. These datasets are often not publicly available and must be purchased or licensed. However, many offer superior spatial and temporal resolution and broader population coverage than open datasets. For instance, mesh-level dwell population data collected by mobile carriers provides expansive geographic coverage (DOCOMO Insight Marketing Inc.). Location datasets gathered via app SDKs vary by app and region, but some offer population coverage ranging from a few to tens of percent (Factori; 4shared; Quadrant Global, 2025). While positioning frequency may vary by user, recent datasets allow reconstruction of detailed movement trajectories updated every few seconds to minutes (GeoTechnologies, Inc.). These rich datasets enable individual-level contact estimation, supporting more advanced modeling capabilities.

A list of representative proprietary datasets is shown in Table 3. Compared to open data, they typically offer higher spatial and temporal coverage, more up-to-date data, and larger data volumes. Despite their benefits, proprietary mobility datasets face three systemic limitations:

1. **k-Anonymity**: This technique groups trajectories so that individuals are indistinguishable within a cluster. While effective in dense urban areas, it performs poorly in low-density regions (de Montjoye et al., 2013).
2. **Differential Privacy**: This method adds noise to ensure mathematically defined privacy guarantees. However, tuning the balance between privacy and data utility is challenging (Abowd, 2018; Dwork & Roth, 2014).
3. **Trajectory Perturbation**: This strategy spatially or temporally modifies trajectory points while maintaining aggregate mobility flows. It has shown promise in preserving both privacy and analytical fidelity, particularly in rural contexts (Athey et al., 2021; de Montjoye et al., 2013).

**Table 3:** Examples of proprietary mobility datasets for epidemiological simulations.

Provider (Dataset)	Region	Period	Spatial Accuracy	Temporal Resolution	Coverage^a^	Demographic Bias	Key Simulation Parameters
Factori Mobility	248 countries	2 yr	GPS (5–10m)	15-min intervals	1–15%∗	Skews 18–55	Dwell-time analytics; Cross-border flows
4shared-Raw	Latam, APAC	2019+	SDK (20–100m)	5-min updates	3–8%	Urban bias	Venue mapping; Activity classification
Quadrant Global	249 countries	2 yr	Cell + WiFi (50m)	Hourly traces	2–10%∗	Low elderly	Origin–dest. matrices; Staypoint detection
Onemata Mobile	212 countries	18 mo	GPS (10m)	30-sec bursts	0.5–5%∗	High-income bias	Speed/direction logs; Transportation mode
Singlespot	France, Belgium	1 yr	BT/WiFi (1m)	2-min pings	12%	Mall/transit users	Indoor positioning; Crowd-density maps
Irys Real-Time	190 countries	6 mo	GPS (5m)	1-sec streams	0.1–3%∗	Delivery drivers	Micro-mobility patterns; Contact-duration metrics
Cuebiq	US-focused	2016+	GPS + SDK (8m)	5-min samples	5–10% US	Rural underrepresented	Census-tract flows; Social distancing scores
SafeGraph Patterns	Global (US)	2018+	POI (1m)	Weekly aggregates	45M POIs	Commercial visits	Foot-traffic analytics; Brand-specific mobility
X-Mode/Digital Envoy	Global	2 yr	SDK (15m)	10-min intervals	1–4%∗	App-dependent	Anomaly detection; Mobility network graphs
Veraset	United States	2019+	GPS (3m)	Continuous	7% US	Android dominance	County-to-county flows; Staypoint clustering

a Coverage refers to estimated percent of the population represented in the dataset.

To mitigate these risks, hybrid modeling approaches have emerged. For example, population re-weighting techniques that combine mobility data with census demographics (Singlespot) help address sampling bias. Federated learning architectures allow privacy-preserving analytics without direct data sharing (Castro Elizondo et al., 2022). Additionally, synthetic data generators trained on proprietary patterns (e.g., SafeGraph POI flows) can simulate realistic contact networks while preserving privacy through controlled noise injection.

Nevertheless, a significant risk remains: data access asymmetry may create a “two-tiered” research ecosystem where commercial players shape public health modeling more than independent researchers. Transparent documentation of dataset limitations, as shown in the demographic bias column in Table 3, and open benchmarking against public surveillance data are necessary to maintain scientific rigor and equity.

### 3.3 Pseudo-world data

Real mobility data—whether open or commercial—faces challenges in spatiotemporal resolution, coverage, accessibility, and privacy. These limitations hinder its direct application in infectious disease propagation modeling, particularly for estimating individual- or facility-level contacts. To address this, synthetic data generation methods that emulate real-world mobility patterns are gaining traction.

For instance, Arcolezi et al. (2020) created synthetic datasets modeling visitor counts during events, distinguishing between first-time and repeat visitors over defined periods. This level of detail is useful for simulating infection risk in crowded environments. Similarly, Kashiyama et al. (2017) generated pseudo human flow data for urban populations by integrating population distributions, trip records, and transit schedules—validating their output against real-world traffic volumes and population densities at 500-m grid intervals. Their approach, while developed for traffic simulations, demonstrates potential applicability for infectious disease modeling.

Advancing the field, Alessandretti et al. (2020) proposed a deep generative model that synthesizes mobility trajectories while preserving spatiotemporal entropy and scaling laws observed in empirical data. Likewise, Song et al. (2020) employed reinforcement learning to generate mobility patterns aligned with urban activity rhythms. However, reliance on biased training data (e.g., GPS logs skewed toward smartphone users) can introduce systematic distortions in the resulting synthetic datasets. Klemmer et al. (2021) addressed validation concerns by comparing synthetic mobility networks with real-world contact patterns during epidemics, revealing trade-offs between privacy and epidemiological fidelity.

These studies collectively demonstrate the potential of synthetic mobility data to bypass privacy concerns by decoupling from real trajectories. However, limitations remain. Facility-level accuracy, for instance, is often poor—many models fail to capture micro-scale interactions such as indoor movement in hospitals. Additionally, source data biases (e.g., smartphone user overrepresentation) or oversmoothing in generation algorithms may distort movement patterns, compromising epidemiological conclusions. Recent releases have begun to address the scarcity of publicly available synthetic datasets: Yuan et al. (2026) released WorldMove, offering city-scale synthetic trajectories across 179 countries, and Kashiyama et al. (2024) developed Pseudo-PFLOW, which reconstructs individual-level daily mobility for approximately 130 million agents across Japan using open data and limited travel surveys.

**Fig. 2:**
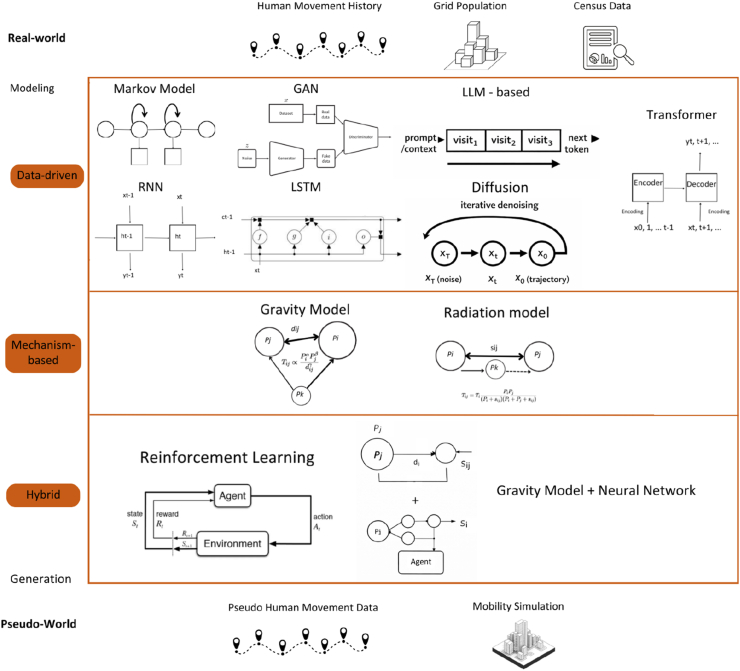
Flow of mobility data modeling, illustrating how real-world data sources are transformed into pseudo-world mobility outputs through data-driven, mechanism-based, and hybrid generation methods.

The overall relationship among real, proprietary, and synthetic mobility data, as well as their roles in epidemiological modeling, is illustrated in Fig. 2.

### 3.4 Pseudo-data generation methods: an overview

The preceding survey revealed that publicly available mobility datasets remain sparse and fragmented, particularly for modeling infection spread at fine spatial and temporal scales. Real-world data are often limited by accessibility, privacy constraints, and inconsistent granularity, which restrict their direct use in high-resolution epidemic simulations. These limitations underscore the need for alternative approaches capable of reproducing realistic movement behaviors while protecting individual privacy. To address this gap, researchers have developed a variety of pseudo-mobility data generation methods that synthetically model human movement based on demographic, behavioral, or geographic priors. Representative approaches—summarized in Table 4—range from classical analytical frameworks such as gravity and radiation models to modern data-driven architectures including agent-based models and deep generative networks.

**Table 4:** Summary of pseudo-mobility data generation methods.

Method	When to Use	Strength	Limitation
Gravity Model	Inter-/intra-regional flows with population and distance data	Simple, interpretable, low data needs	Overestimates short trips; ignores accessibility heterogeneity

Radiation Model	Population/opportunity-based flow estimation	Parameter-free; theory-grounded	Assumes uniform accessibility; weak in urban networks

Agent-Based Model (ABM)	Micro-level contacts; scenario/policy testing	Captures heterogeneity and interactions	Data- and compute-intensive; calibration heavy

Activity-Based Model	Daily routines (home–work–other) with OD/survey inputs	Realistic time-of-day patterns	Requires detailed activity surveys; complex setup

Reinforcement Learning Models	Adaptive behaviors under changing policies	Learns optimal responses; supports counterfactuals	Needs large training data; less interpretable

GANs	High-fidelity synthetic trajectories	Realistic traces; privacy by synthesis	Mode collapse risk; heavy training requirements

RNN/LSTM	Sequential trajectory generation	Captures temporal dependencies	Struggles with sparsity/irregular sampling

Transformer-Based Models	Long sequences, citywide sparse data	Handles long-range correlations	High computational cost; overfitting risk

Trajectory Perturbation	Privacy-preserving releases with path realism	Preserves aggregates/flows	Distorts fine-scale contacts

Differential Privacy	Strong privacy compliance (e.g., GDPR)	Formal privacy guarantees	Noise reduces accuracy/utility

Hybrid (e.g., Gravity + ABM)	Multi-scale needs (regional + local)	Combines interpretability and realism	Complex calibration/validation

Synthetic Population Generators	Regions lacking microdata/census detail	Builds realistic demographics/households	Sensitive to input bias; may miss behaviors

Federated Learning Simulations	Data cannot leave devices/providers	Privacy-preserving decentralized training	Infrastructure required; slower convergence

Diffusion Models	Trajectory recovery; OD matrix generation from imagery or aggregated data	High sample diversity; strong distributional fidelity	Slow sampling; large memory footprint

Autoregressive Models	Token-level trajectory synthesis with travel-pattern conditioning	Captures sequential regularities; flexible conditioning	Error accumulation over long horizons

Because the primary objective of this paper is to survey mobility *datasets* rather than generation *algorithms*, this section provides a concise methodological overview intended to contextualize the synthetic datasets identified above and to guide future data-production efforts, rather than offering an exhaustive treatment of the generation literature. Readers seeking comprehensive reviews of generative mobility models are referred to dedicated surveys on trajectory generation (Zhu et al., 2025) and origin–destination estimation (Rong et al., 2024a).

Radiation models offer a parameter-free alternative based on opportunity theory (Simini et al., 2012a). These models assume that individuals select the closest location that satisfies their needs, with likelihoods based on surrounding population densities. Although they reproduce long-tailed travel distributions, they often assume uniform accessibility—an unrealistic condition in many real-world scenarios (Kang et al., 2022; Simini et al., 2012b).

Modern data-driven methods enhance these classical approaches by integrating them with empirical datasets. Activity-based models simulate daily routines by chaining locations (e.g., home → work → shopping) based on origin-destination matrices, census data, and mobile logs (McNally, 2007, pp. 55–73). Agent-based models (ABMs) go further by simulating autonomous agents influenced by demographic attributes, social interactions, and environmental cues. ABMs can incorporate gravity or radiation models for macro-flow generation while using reinforcement learning (RL) to model adaptive behaviors like route changes or responses to policy interventions (Pang et al., 2018, 2020).

Emerging hybrid methods employ machine learning architectures such as generative adversarial networks (GANs), recurrent neural networks (RNNs), long short-term memory (LSTM), and Transformer-based models. These frameworks synthesize fine-grained mobility trajectories (Chen, Hu, et al., 2020; Feng et al., 2020; Liu et al., 2016; Ouyang et al., 2018; Yuan et al., 2022). GANs create realistic movement traces, LSTMs capture temporal dependencies, and Transformers effectively model long-range correlations in sparse datasets. Often, classical models are embedded as priors—for example, gravity models constrain spatial distributions, helping neural networks generate geographically plausible outputs (Cheng et al., 2023; Lin et al., 2021). This integration enables the simulation of region-wide mobility as well as individual-level patterns, accounting for periodic behaviors and context-dependent variations.

More recently, diffusion-based and autoregressive generative models have attracted considerable attention in the computer science community for both trajectory synthesis and origin–destination (OD) flow generation. Diffusion models iteratively denoise random samples to produce realistic mobility sequences, and have been applied to individual trajectory recovery (Long et al., 2025b), group mobility generation (Long et al., 2025a), and large-scale OD matrix synthesis from satellite imagery (Rong et al., 2023; Wang, Zhao, et al., 2025). Autoregressive approaches, which generate trajectories token-by-token conditioned on preceding steps, have shown strong performance in capturing travel-pattern regularities (Luo et al., 2025). Large language models (LLMs) have also been explored as mobility generators, treating trajectory prediction as a sequence-modeling task; notable examples include LLMob (Wang et al., 2024), which frames personal mobility generation as an LLM agent problem, and MobGLM (Zhang et al., 2024), which fine-tunes a language model on tokenized GPS traces. Hybrid frameworks that combine mechanistic priors (e.g., gravity constraints) with learned neural components have also been proposed, such as Act2Loc (Liu et al., 2024), which couples activity-chain models with machine learning to achieve both interpretability and fine-grained fidelity. Deep learning pipelines that ingest aggregated mobility statistics or remote-sensing imagery to predict population flows represent another promising direction (Simini et al., 2021; Xu et al., 2025). A dedicated benchmark dataset for commuting OD generation has also been recently released, facilitating standardized evaluation of these methods (Rong et al., 2024b).

While each approach has its strengths, they also come with limitations. Classical models are interpretable but cannot reflect individual variability. Deep learning methods, though powerful, often require extensive training data and lack interpretability. Hybrid approaches attempt to combine their advantages, yet technical challenges remain in calibration, validation, and maintaining coherence across spatial scales. A comparative overview of the major pseudo-mobility data generation methods is presented in Table 4, which summarizes their typical use cases, key strengths, and limitations. No single approach is universally optimal; their suitability depends on data availability, spatial scale, and modeling objectives. Integrating classical and AI-based techniques can enable multi-scale mobility simulations that support epidemic preparedness, transportation optimization, and data-driven urban planning.

Given the rapid pace of development in generative mobility modeling, a systematic review of these approaches—including benchmarking of generation quality and downstream epidemiological utility—is beyond the scope of this dataset-focused survey and constitutes an important direction for a dedicated future review article.

## 4 Discussion

### 4.1 Problems of existing datasets and generation methods

The utility of human mobility data in the modeling of infectious diseases is counterbalanced by three major limitations in existing datasets. First, real-world datasets exhibit notable geographical and demographic biases. Rural areas and economically disadvantaged regions are often underrepresented due to lower mobile device penetration rates (Buckee et al., 2020; Wesolowski et al., 2019; Zagheni et al., 2017). Similarly, elderly populations, minority communities, and low-income households are less represented due to disparities in device ownership and digital access. These gaps reduce the model's ability to predict disease spread among vulnerable groups.

Second, there are significant resolution trade-offs. GPS data provides high spatial resolution but limited temporal resolution due to constraints in battery life, data storage, and cost (Jiang et al., 2013; Pappalardo et al., 2015). Most datasets rely on hourly or daily aggregates, which overlook critical short-duration contacts. Moreover, indoor mobility and movement across different floors in buildings remain poorly captured (Wijayarathna & Aickelin, 2018), resulting in blind spots for contact tracing efforts.

Third, accessibility barriers limit the practical use of mobility data. While large commercial datasets exist, public access is restricted due to privacy regulations like GDPR and proprietary business interests (European Union, 2016; Oliver et al., 2020). Even when available, such data is often heavily aggregated to maintain anonymity, which compromises its analytical granularity (Berke et al., 2019; Zang & Bolot, 2011, pp. 145–156). Although the COVID-19 pandemic led to temporary increases in data availability, sustainable open-access mechanisms remain scarce (Oliver et al., 2020).

In this study, pseudo-data is defined as mobility data generated through modeling techniques—such as agent-based simulations, GANs, or rule-based approaches—that emulate aggregate human movement patterns. This is distinct from enhanced real-world data generated through interpolation or super-resolution.

Synthetic population modeling addresses some limitations (Athey et al., 2021; Beckman et al., 1996), offering privacy-preserving datasets that simulate mobility trends. However, pseudo-data also faces challenges: it often lacks the spatial resolution needed for individual-level modeling and is typically aggregated to Points of Interest (POIs) or base station coverage zones. Moreover, the reliance on biased training data may carry those biases into the synthetic data. High-resolution pseudo-data may even risk re-identification if it replicates real user behavior too closely (Rocher et al., 2019). Finally, pseudo-data temporal resolution remains tied to that of the original real-world datasets (Pappalardo et al., 2015), limiting its ability to model short-term contact events.

### 4.2 Validation and evaluation

Travel surveys are essential for validating both real and synthetic mobility datasets. By comparing model outputs with ground-truth survey metrics such as visitor volumes, contact probabilities, and inter-zonal flows, researchers can detect biases and calibration errors. Regional surveys like the MWCOG Household Travel Survey (Metropolitan Washington Council of Governments, 2021) offer demographic-stratified data for validation, while urban-level surveys such as the NYC DOT Citywide Mobility Survey (New York City Department of Transportation, 2022) provide insight into spatial coverage.

For infectious disease modeling, validation must occur at resolutions meaningful to epidemiology. Integrating vertical mobility data from barometer and accelerometer sensors (Issawy et al., 2023) enhances contact tracing accuracy in multi-floor buildings. However, survey-based validation is limited by temporal mismatches: most surveys provide only annual data, while mobility patterns can shift daily.

### 4.3 Anonymity and privacy preservation

Synthetic data generation is increasingly viewed as a solution for both privacy protection and bias mitigation (Athey et al., 2021). By decoupling data from real individual trajectories, it circumvents strict anonymization protocols such as those mandated by the GDPR (European Union, 2016). However, high-fidelity generation can still encode real-world behavioral patterns, especially in sparsely populated areas, raising re-identification concerns (de Montjoye et al., 2013; Rocher et al., 2019).

Three main approaches aim to balance privacy and data utility:

k-Anonymity: This technique groups trajectories so that individuals are indistinguishable within a cluster. While effective in dense urban areas, it performs poorly in low-density regions (de Montjoye et al., 2013).

Differential Privacy: This method adds noise to ensure mathematically defined privacy guarantees. However, tuning the balance between privacy and data utility is challenging (Abowd, 2018; Dwork & Roth, 2014).

Trajectory Perturbation: This strategy spatially or temporally modifies trajectory points while maintaining aggregate mobility flows. It has shown promise in preserving both privacy and analytical fidelity, particularly in rural contexts (Athey et al., 2021; de Montjoye et al., 2013).

Hybrid techniques, such as aggregating GPS data into 1 km^2^ grids combined with trajectory swapping, have proven effective in low-density areas (Athey et al., 2021; de Montjoye et al., 2013). While such methods reduce fine-grained realism, they maintain utility for macro-level epidemiological modeling (Pappalardo et al., 2015). Balancing this privacy-utility tradeoff is particularly challenging in cases where the same data must serve both individual-level contact tracing and regional policy design.

## 5 Concluding remarks

This survey confirms that mobility and behavior data available for infectious disease simulation remain limited. This highlights the need for improving accessibility and developing new synthetic data generation techniques. To address limitations in the spatial and temporal resolution of existing mobility datasets, an integrated modeling approach combining traditional census data with high-resolution mobile data (e.g., GPS, CDRs) offers strong potential. Census data provides comprehensive demographic coverage, while mobile datasets capture detailed spatiotemporal behaviors. The fusion of these datasets enables the construction of rich, multi-layered mobility networks that mitigate the weaknesses of each source.

This approach has been applied effectively in contexts such as modeling the Ebola outbreak in West Africa, where combining CDR and census data enhanced predictions of epidemic trajectories (Wesolowski et al., 2014). Nonetheless, calibration remains essential to correct for known biases—particularly the overrepresentation of urban and higher-income populations in mobile data (Chen & Kumar, 2018; Garcia & Patel, 2017; Johnson & Lee, 2019).

Another emerging opportunity lies in the development of privacy-preserving generative models. Frameworks such as MoGAN and PateGail, based on GANs and federated learning respectively, allow the creation of synthetic datasets that mirror statistical properties of real data without exposing sensitive individual trajectories (Kim & Roberts, 2021; Wang & Liu, 2020). These methods also scale well to large populations and global settings. In regions lacking sufficient training data, transfer learning techniques can adapt pre-trained generative models to local conditions (Martinez & Singh, 2022). As foundational modeling, validation, and data governance frameworks continue to evolve, these innovations hold promise for enabling broader, fairer, and more secure access to high-quality mobility data in support of public health modeling and urban resilience.

The contributions of this paper are summarized as follows. First, we provide a structured inventory of the latest real-world and synthetic (pseudo) mobility and location datasets that are beneficial for modeling the transmission and spread of infectious diseases. Second, we offer a concise overview of pseudo-mobility data generation methods to contextualize the synthetic datasets and guide future data-production efforts; we note that the rapid development of generative approaches—particularly diffusion-based, autoregressive, and LLM-based models—warrants a dedicated review article for a comprehensive and technically detailed treatment. Finally, we identify key challenges associated with existing mobility data—including geographic bias, resolution trade-offs, and accessibility barriers—and discuss potential future directions. Collectively, these findings provide guidance for researchers and policymakers in selecting or developing mobility datasets that support effective modeling of outbreaks and design of intervention strategies.

## CRediT authorship contribution statement

**Nodira Tillayeva:** Writing – original draft, Software, Methodology, Investigation, Data curation. **Naoki Tamura:** Writing – original draft, Investigation, Data curation. **Kenta Urano:** Writing – review & editing, Supervision. **Takuro Yonezawa:** Writing – review & editing, Supervision, Project administration. **Nobuo Kawaguchi:** Writing – review & editing, Supervision, Funding acquisition. **Takashi Okumura:** Writing – review & editing, Supervision, Project administration, Conceptualization.

## Code availability

The code used for the systematic literature review and large language model (LLM)–based analysis is available from the corresponding author upon reasonable request.

## Funding

This work was supported by the National Institute of Information and Communications Technology (NICT) under Grant No. 222C01, and by the Japan Science and Technology Agency under Grant Nos. JPMJCR21F2 and JPMJCR22M4.

## Conflict of interest

The authors have declared no conflict of interest.

## Figures and Tables

**Table A.1:** Summary of publicly available human mobility datasets with full citation references (Part 1: Aggregated Index, Mesh-Level, and OD Trip Data)

No.	Data Category	Name & Provider	Region	Period	Spatial Resolution/# Locations	Temporal Resolution/# Records
1	Administrative Division Level Statistics and Index Data	Google LLC∗∗Google LLC., 2020	Worldwide	2020/02/15–2022/10/15	City, Country, State	1 Day
2		Apple Mobility Data∗∗Apple Inc., 2020	Global	2020/01/13–2022/04/14	City, Region, Country	1 Day
3		Baidu Mobility Index Baidu Inc., 2020	China	2020/03/15–present	City, Province	1 Day
4		Descartes Lab Mobility Index Davis	U.S.	2020/03/01–2021/04/20	State, Country	1 Day
5		University of Maryland Mobility Metrics, Zhang et al. (a)	U.S.	2020/01/01–2021/04/20	State	1 Day
6		ODT Flow Explorer, Li et al. (2021a)	U.S.	2019/01/01–2021/04/15	State, Country	1 Day
7		Tencent Migration Data Qianxi Tencent Holdings Ltd.	China	2020/01/01–present	City, Province	1 Day
8		SafeGraph Social Distancing Metrics∗∗SafeGraph Inc., 2020 (now Advan)	U.S.	2020/03/01–2022/12/31	State, County	1 Day
9		Cuebiq Data for Good Cuebiq Inc., 2020	Global	2020/01/01–present	Administrative Divisions	1 Day
10		Meta Data for Good Meta Platforms Inc.	Global	2020/01/01–present	Administrative Divisions	1 Day
11		BBC Pandemic Model Data, Klepac et al. (2018)	UK	2018/01/01–2019/12/31	City, Region	Survey Period
12		GLEAMviz H1N1 Mobility Balcan et al.	Global	2009/01/01–2010/12/31	Country	Modeled Daily
13		Unacast Social Distancing Dataset∗∗Walle	Global	2020/03/01–2021/12/31	Administrative Divisions	1 Day
14		MobMeter (Finazzi/Univ. Bergamo) Finazzi (2023)	17 countries	2020/03–present	Country, Subnational	1 Day
15	Mesh Level Mobility Data	Telecommunications Data Telecom Italia (2015)	Milan, Italy	2013/11/01–2014/01/01	250 m Mesh	Hourly
16		WorldPop Dynamic Population WorldPop	France, Portugal	2007/05/01–2007/10/31	100 m Mesh	1 Day
17		Yahoo JAPAN Congestion Ladder Yahoo! JAPAN Corporation, 2020	Japan	Historical (1 Day)	100 m Mesh	Hourly
18		NTT docomo Mobile Spatial Statistic DOCOMO Insight Marketing Inc.	Japan	Historical (1 Day)	500 m Mesh	Hourly
19		D4D Orange Telecom Blondel et al. (2013)	Côte d’Ivoire, Senegal	2013/01/01–2013/12/31	500 m Mesh	1 Day
20		LYMob-4Cities Yabe et al.	Japan	2020/01/01–present	100 m Mesh	Hourly
21		dtac Mobility Data Total Access Communication Public Company Limited (DTAC)	Thailand	2020/03/01–present	1 km Mesh	1 Day
22		YJMob100K (Yahoo Japan/LY Corp.) Yabe et al. (2024)	Japan	75 days (60 + 15 emergency)	500 m Mesh	30 min; 100K users
23	OD Trip Data	CMAP Travel Survey Chicago Metropolitan Agency for Planning	U.S.	2017/10/02–2019/01/20	110 k Locations	98 k Records
24		MITMA Daily OD Ministerio de Transportes, Movilidad y Agenda Urbana (MITMA), 2020	Spain	2020/01/01–present	Municipalities	1 Day
25		NYC Taxi Trips New York City Taxi and Limousine Commission, 2020	NYC, U.S.	2009/01/01–present	Taxi Zones	Trip-based
26		Chicago Divvy Bike City of Chicago Department of Transportation and Lyft, 2020	Chicago, U.S.	2013/06/01–present	Stations	Trip-based
27		DiDi GAIA Didi Chuxing GAIA Initiative, 2018	Chengdu, China	2018/11/01–2018/11/30	Road Segments	2–4 s
28		Global Airline Traffic International Air Transport Association (IATA), 2020	Global	2019/01/01–present	Airports	1 Day
29		MOBINS-Epidemic-Korea (KAIST DMLAB) Na et al. (2025)	Korea (Seoul, Busan, Daegu)	COVID-19 pandemic period	City, District	1 Day
30		MOBINS-Epidemic-NYC (KAIST DMLAB) Na et al. (2025)	NYC, U.S.	COVID-19 pandemic period	Borough, Zip	1 Day
31		US Census LODES/LEHD U.S. Census Bureau (2024)	U.S.	2002–present (annual)	Census Block, Tract	Annual; ∼7M OD pairs

Full citation keys refer to Table A2 for complete reference entries.

∗∗Datasets have ceased updating or are no longer publicly available.

**Table A.2:** Summary of publicly available human mobility datasets with full citation references (Part 2: Point-Level Movement Data)

No.	Data Category	Name & Provider	Region	Period	Spatial Resolution/# Locations	Temporal Resolution/# Records
32	Point-Level Movement Data	AnLoCOV, Moncayo-Unda et al. (2022)	Ecuador	2013/06/21–2022/04/05	Point (Latitude, Longitude)	16M Records
33		MOBIS, Molloy et al. (2022)	Switzerland	2019/09/01–2020/02/29		3M Records
34		Shanghai GPS Data, Zhou (2021)	Shanghai, China	2013/10/18–2015/11/25		10k Records
35		NREL California Survey National Renewable Energy Laboratory, 2013	California, U.S.	2012/02/01–2013/02/03		3M Records
36		CRAWDAD EPFL Taxi, Piorkowski et al. (2022)	San Francisco, U.S.	2008/05/17–2008/06/10		112M Records
37		Copenhagen Networks Study Copenhagen Networks Study, 2025	Denmark	2013/01/01–2014/12/31		1M Records∗
38		MIT Reality Mining Eagle and Pentland, 2006	Boston, U.S.	2004/09/01–2005/06/30		1M Records∗
39		Geotagged Tweets, Hawelka et al. (2014)	Global	2020/01/01–present		1B + Records
40		OpenStreetMap GPS OpenStreetMap Contributors, 2005	Global	2005/01/01–present		50M Records∗
41		Iquitos GPSS, Schaber et al.	Peru	2018/01/01–2019/12/31		150k Records
42		LBSLab WeChat, Zhang et al. (b)	China	2020/03/01–present		10M Records
43		Geolife GPS Trajectories, Zheng et al.	Beijing, China	2007/04/01–2012/08/31		1–5 Seconds
44		CRAWDAD ROMA/TAXI, Bracciale et al. (2022)	Rome, Italy	2014/02/01–2014/03/02		7 Seconds
45		ECML/PKDD 15 Taxi, Moreira-Matias et al., 2015	Porto, Portugal	2013/01/07–2014/06/30		15 Seconds
46		TaxiBJ21 Jiang (2022)	Beijing, China	2012/11/01–2015/11/30 (3 months)		1 Minute
47		Mobile Data Challenge, Laurila et al. (2012)	Switzerland	2009–2011		1–5 Seconds
48		T-Drive, Zheng (2011)	Beijing, China	2008/02/02–2008/02/08		15M Records
49		Brightkite Check-in Cho et al. (2011a)	Global	2008/04/01–2010/10/31		4.5M Records
50		Gowalla Check-in Cho et al. (2011b)	Global	2009/02/01–2010/10/31		6.4M Records
51		GeoCOV19Tweets Lamsal, 2021	Global	2020/03/20–2023/03/15		502k Records
52		MegaGeoCOV Lamsal et al., 2022	Global	2020/01/01–2021/09/16		25M Records

Full citation keys refer to Table A.2 for complete reference entries.

∗Approximate values.

**Table A.3:** Dataset Number, Name, and Full Reference (Part 1)

No.	Dataset Name	Reference (formatted in Elsevier style)
1	Google LLC	Google LLC. (2020). COVID-19 Community Mobility Reports. https://www.google.com/covid19/mobility/
2	Apple Mobility Data	Apple Inc. (2020). Mobility Trends Reports. https://covid19.apple.com/mobility
3	Baidu Mobility Index	Baidu. (2020). Baidu Mobility Data. https://qianxi.baidu.com/
4	Descartes Lab Mobility Index	Descartes Labs. (2020). Mobility Index during COVID-19. https://www.descarteslabs.com/mobility/
5	University of Maryland Mobility Metrics	University of Maryland. (2021). COVID-19 Impact Analysis Platform. https://data.covid.umd.edu/
6	ODT Flow Explorer	Kang, Y. et al. (2022). Multiscale Mobility Networks and the Spatial Spreading of Infectious Diseases. *PNAS*, 117(26), 14857–14863. https://covid19-mobility.netlify.app/
7	Tencent Migration Data Qianxi	Tencent. (2020). Qianxi Migration Data. https://heat.qq.com/
8	SafeGraph Social Distancing Metrics	SafeGraph Inc. (2020). Social Distancing Metrics. https://www.safegraph.com/covid-19-data-consortium
9	Cuebiq Data for Good	Cuebiq. (2020). Mobility Data for Good. https://www.cuebiq.com/about/data-for-good/
10	Meta Data for Good	Meta. (2020). Data for Good Program. https://dataforgood.facebook.com/
11	BBC Pandemic Model Data	BBC. (2019). BBC Pandemic project: Human mobility and infection modeling data. https://www.bbc.co.uk/mediacentre/latestnews/2018/pandemic
12	GLEAMviz H1N1 Mobility	Van den Broeck, W. et al. (2011). The GLEAMviz computational tool, a publicly available software to explore realistic epidemic spreading scenarios at the global scale. *BMC Infectious Diseases*, 11(1), 37. https://www.gleamviz.org/
13	Unacast Social Distancing Dataset	Unacast. (2020). COVID-19 Social Distancing Scoreboard. https://www.unacast.com/covid19/social-distancing-scoreboard
14	MobMeter	Finazzi, F. (2023). Replacing discontinued Big Tech mobility reports: a penetration-based analysis. *Scientific Reports*, 13, 1271. https://doi.org/10.1038/s41598-023-28137-7
15	Telecommunications Data	Toole, J. L. et al. (2015). Tracking anonymized mobile phone data to improve urban planning. *PNAS*, 112(22), 6823–6828.
16	WorldPop Dynamic Population	Stevens, F. R. et al. (2015). Disaggregating census data for population mapping using Random Forests with remotely-sensed and ancillary data. *PLOS ONE*, 10(2), e0107042. https://www.org/WorldPop
17	Yahoo JAPAN Congestion Ladder	Yahoo Japan Corporation. (2020). Congestion prediction and mobility visualization service. https://about.yahoo.co.jp/pr/release/2020/03/27a/
18	NTT docomo Mobile Spatial Statistic	NTT Docomo. (2020). Mobile spatial statistics. https://www.nttdocomo.co.jp/corporate/disclosure/mobile_spatial_statistics/
19	D4D Orange Telecom	Blondel, V. D. et al. (2013). Data for Development: The D4D Challenge on Mobile Phone Data. https://www.d4d.orange.com
20	LYMob-4Cities	Yabe, T. et al. (2022). Cross-comparative analysis of mobility patterns during COVID-19 using large-scale GPS data. *Nature Communications*, 13(1), 4253.
21	dtac Mobility Data	dtac. (2020). Human mobility dataset for Thailand during COVID-19. https://covid-mobility.dtac.co.th
22	YJMob100K	Yabe, T. et al. (2024). YJMob100K: City-scale and longitudinal dataset of anonymized human mobility trajectories. *Scientific Data*, 11, 397. https://doi.org/10.1038/s41597-024-03237-9
23	CMAP Travel Survey	Chicago Metropolitan Agency for Planning. (2020). Travel Tracker Survey Dataset. https://www.cmap.illinois.gov/data/transportation/travel-tracker-survey
24	MITMA Daily OD	Ministerio de Transportes, Movilidad y Agenda Urbana (MITMA). (2020). Daily Mobility Studies. https://www.mitma.gob.es/
25	NYC Taxi Trips	NYC TLC. (2020). Taxi and Limousine Commission Trip Record Data. https://www1.nyc.gov/site/tlc/about/tlc-trip-record-data.page
26	Chicago Divvy Bike	Divvy Bikes. (2020). Chicago Bike Share System Data. https://divvybikes.com/system-data
27	DiDi GAIA	DiDi Chuxing. (2018). DiDi GAIA Open Data Initiative. https://outreach.didichuxing.com/research/opendata/
28	Global Airline Traffic	International Air Transport Association (IATA). (2020). Global Airline Movement Database. https://www.iata.org/
29	MOBINS-Epidemic-Korea	Na, J. et al. (2025). MOBINS: Mobility-Integrated Benchmark for Infectious Disease and Network Surveillance. Zenodo. https://doi.org/10.5281/zenodo.14590709
30	MOBINS-Epidemic-NYC	Na, J. et al. (2025). MOBINS: Mobility-Integrated Benchmark for Infectious Disease and Network Surveillance. Zenodo. https://doi.org/10.5281/zenodo.14590709
31	US Census LODES/LEHD	U.S. Census Bureau. (2024–present). LEHD Origin-Destination Employment Statistics (LODES). https://lehd.ces.census.gov/data/
32	AnLoCOV	Sulyok, M., Walker, M. (2020). Community mobility in Ecuador and COVID-19 transmission. *Scientific Reports*, 10, 19130.
33	MOBIS	Becker, F. et al. (2020). MOBIS-Covid19: A panel survey dataset on mobility behavior. https://ivtmobis.ethz.ch/mobis/covid19/
34	Shanghai GPS Data	Zheng, Y. et al. (2011). Urban computing with taxicabs. In: Proceedings of UbiComp.
35	NREL California Survey	National Renewable Energy Laboratory (NREL). (2013). California Household Travel Survey. https://www.nrel.gov/
36	CRAWDAD EPFL Taxi	CRAWDAD. (2009). EPFL Taxi dataset. https://crawdad.org/epfl/mobility/20090224
37	Copenhagen Networks Study	Stopczynski, A. et al. (2014). Measuring large-scale social networks with high-resolution data. *Scientific Reports*, 4, 6174.
38	MIT Reality Mining	Eagle, N., Pentland, A. (2006). Reality mining: sensing complex social systems. *Personal and Ubiquitous Computing*, 10(4), 255–268.
39	Geotagged Tweets	Hawelka, B. et al. (2014). Geo-located Twitter as proxy for global mobility patterns. *Cartography and Geographic Information Science*, 41(3), 260–271.
40	OpenStreetMap GPS	OpenStreetMap Contributors. (2005–2023). GPS Trace Dataset. https://www.openstreetmap.org/traces
41	Iquitos GPS	Perkins, T. A. et al. (2020). Intra-urban mobility and dengue incidence in Iquitos, Peru. *PLoS Neglected Tropical Diseases*, 14(3): e0008123.
42	LBSLab WeChat	Yuan, N. J. et al. (2016). Discovering urban functional zones using mobility data from WeChat. In: Proceedings of WWW 2016.
43	Geolife GPS Trajectories	Zheng, Y. et al. (2009). Geolife: A collaborative social networking service using GPS trajectories. *IEEE Data Engineering Bulletin*, 33(2), 32–39.
44	CRAWDAD ROMA/TAXI	CRAWDAD. (2014). Roma Taxi dataset. https://crawdad.org/roma/taxi/20140717
45	ECML/PKDD 15 Taxi	Moreira-Matias, L. et al. (2015). Predicting taxi–passenger demand using streaming data. *ECML/PKDD Taxi Dataset*.
46	TaxiBJ21	Zhang, J. et al. (2017). Deep Spatio-Temporal Residual Networks for Citywide Crowd Flows Prediction. In: Proceedings of AAAI. Dataset: TaxiBJ. https://github.com/TolicWang/DeepST
47	Mobile Data Challenge	Laurila, J. K. et al. (2012). The Mobile Data Challenge: Big data for mobile computing research. In: Nokia Mobile Data Challenge.
48	T-Drive	Yuan, J. et al. (2011). T-Drive: Driving directions based on taxi trajectories. In: Proceedings of SIGSPATIAL. Dataset: T-Drive. https://www.microsoft.com/en-us/research/publication/t-drive-driving-directions-based-on-taxi-trajectories/
49	Brightkite Check-in	Cho, E. et al. (2011). Friendship and mobility: User movement in location-based social networks. In: Proceedings of KDD. Dataset: Brightkite.
50	Gowalla Check-in	Cho, E. et al. (2011). Friendship and mobility: User movement in location-based social networks. In: Proceedings of KDD. Dataset: Gowalla.
51	GeoCOV19Tweets	Lamsal, R. (2021). Design and analysis of a large-scale COVID-19 tweets dataset. *Applied Intelligence*, 51(5), 2790–2804. https://crisisnlp.qcri.org/covid19
52	MegaGeoCOV	Lamsal, R. (2022). MegaGeoCOV: Global Geotagged Twitter Dataset on COVID-19. *Data in Brief*, 40, 107819. https://doi.org/10.1016/j.dib.2022.107819

**Figure:**
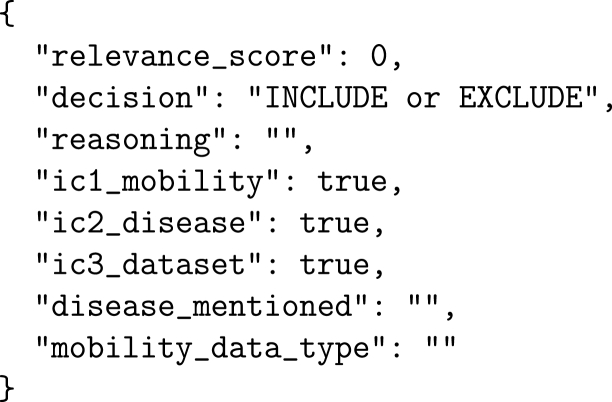


**Figure:**
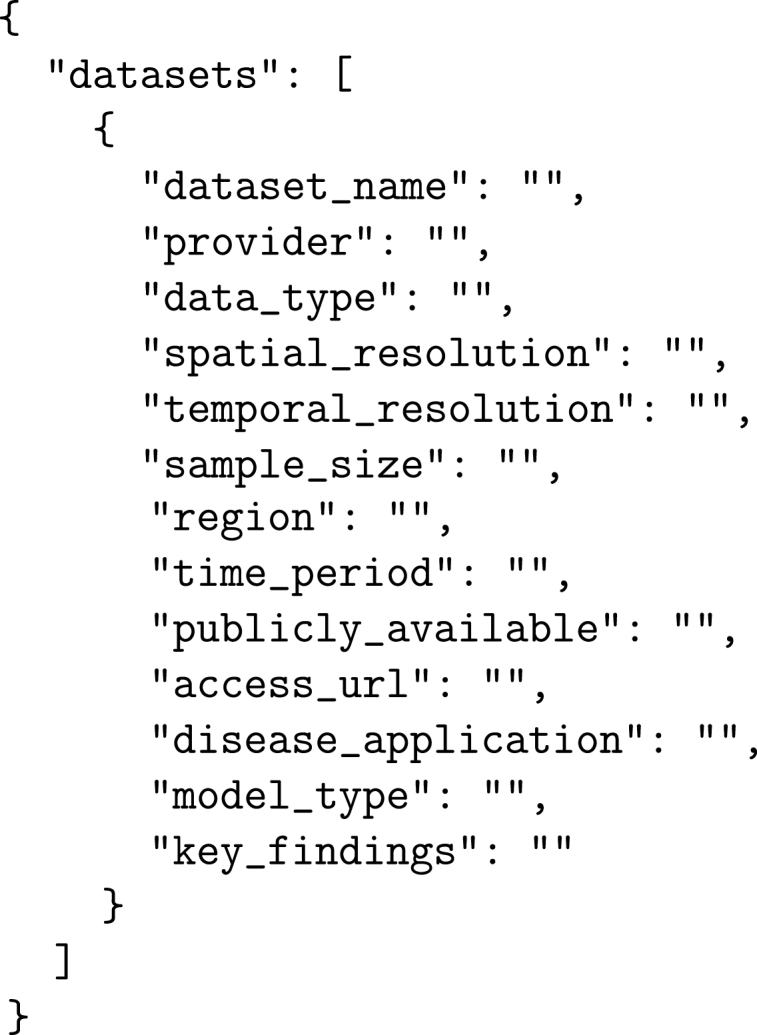


## Data Availability

All datasets referenced in this study can be accessed as described in the cited publications or via the respective official websites. Open datasets are freely available for download, whereas synthetic and proprietary datasets may require registration, purchase, or licensing from the respective providers.

## Appendix B LLM-Assisted Screening Methodology

To ensure transparency and reproducibility of our screening process, we provide the complete prompts and output schemas used for LLM-assisted abstract screening and data extraction. All prompts were executed with the following model configuration:

• **Model**: GPT-4-turbo (gpt-4-turbo)
• **Temperature**: 0 (deterministic output)
• **Max tokens**: 1024 (Prompt #1), 2048 (Prompt #2)
• **API version**: OpenAI Python SDK v1.12+

### Appendix B.1 Prompt #1: Title/Abstract Screening

#### System:

You are a systematic-review screening assistant for a scoping review about **human mobility datasets used in infectious disease modelling**. Evaluate whether a research paper should be INCLUDED based on its title and abstract.

#### Task:

Analyze the title and abstract provided below and determine whether the paper meets all three inclusion criteria. Use only information explicitly present in the title and abstract; if a criterion cannot be confirmed, treat it as not met. Do not hallucinate information that is not present in the text.

#### **Inclusion criteria** (all three must be met):

• **IC1 (Mobility)**: The paper is substantially about human population movement or mobility (commuting, migration, travel, mobile-phone traces, GPS trajectories, flight networks, etc.).
• **IC2 (Disease)**: The paper relates human mobility to infectious disease transmission, epidemiological modelling, or outbreak response.
• **IC3 (Dataset)**: The paper uses, describes, evaluates, or generates a mobility dataset (not merely mentions mobility as a concept).

#### **Exclusion criteria** (any single criterion triggers exclusion):

• **EC1**: Non-human mobility only (avian migration, livestock movement) with no human mobility component.
• **EC2**: Indoor airflow or ventilation CFD modelling only, with no spatial mobility data.
• **EC3**: Policy commentary or editorial with no original data analysis or dataset description.
• **EC4**: Mobility mentioned only metaphorically or tangentially (e.g., “social mobility”).

#### Scoring rubric (0–10):

• **0**: Completely irrelevant (none of IC1–IC3 met).
• **1**–**2**: Tangentially related; mobility or disease mentioned but not the paper's focus; no dataset.
• **3**–**4**: Partially relevant; covers mobility OR disease, but not both together, or no real dataset.
• **5**–**6**: Moderately relevant; covers mobility and disease but dataset usage is unclear or secondary.
• **7**–**8**: Highly relevant; clearly about human mobility data applied to infectious disease; dataset is used but may not be the paper's main contribution.
• **9**–**10**: Core relevance; the paper's primary focus is a mobility dataset (or generating one) applied to infectious disease modelling.

Decision rule: score ≥5→ include; score <5→ exclude.

#### Few-shot calibration examples:

Three calibration examples were provided in the prompt to anchor scoring:

1. A paper using CDR-derived mobility data to model measles spread (expected score: 9, INCLUDE).
2. A paper on satellite-based air quality changes during lockdowns (expected score: 2, EXCLUDE).
3. A paper forecasting dengue using Google Trends with mobility discussed but not measured (expected score: 3, EXCLUDE).

#### Output schema:

Return exactly one JSON object. Use snake_case keys.

Return only valid JSON conforming exactly to this schema—no markdown, prose, or code-fences.

### Appendix B.2 Prompt #2: Data Extraction

#### System:

You are a data-extraction assistant for a systematic review about **human mobility datasets used in infectious disease modelling**. Follow the JSON schema exactly.

#### Task:

Analyze the full text of the included paper and extract the data points below for *each* mobility dataset mentioned in the paper. Use only information explicitly present in the text; if absent, write “Not Available” (without quotes for numeric fields). Do not hallucinate information that is not present in the paper.

#### Required fields & allowed values:

• **dataset_name** – free text or “Not Available”
• **provider** – free text or “Not Available”
• **data_type** – “CDR” | “GPS” | “Survey” | “Social Media” | “Transit” | “Flight” | “Synthetic” | “Other” | “Not Available”
• **spatial_resolution** – free text (e.g., “country-level”, “500m grid”) or “Not Available”
• **temporal_resolution** – free text (e.g., “hourly”, “daily”, “weekly”) or “Not Available”
• **sample_size** – free text or “Not Available”
• **region** – free text or “Not Available”
• **time_period** – free text (e.g., “2020-01 to 2020-12”) or “Not Available”
• **publicly_available** – “Yes” | “No” | “Partially” | “Not Available”
• **access_url** – URL string or “Not Available”
• **disease_application** – free text or “Not Available”
• **model_type** – free text (e.g., “SIR”, “Agent-based”, “Gravity model”) or “Not Available”
• **key_findings** – one-sentence summary or “Not Available”

#### Output schema:

Return exactly one JSON object containing an array of dataset records. Use snake_case keys.

Return only valid JSON conforming exactly to this schema—no markdown, prose, or code-fences.

#### Appendix B.3 Human Verification

All LLM decisions were subject to human review:

• 20% random sample of INCLUDE decisions were verified.
• All borderline papers (score 4–6) were manually reviewed.
• All EXCLUDE decisions with score >3 were checked for false negatives.
• Dataset extraction accuracy (URLs, names) was verified against original publications.

### Appendix B.4 Reproducibility

The complete Python implementation of the screening pipeline, including all prompts and few-shot examples, is available from the corresponding author upon reasonable request.
